# The Recruitment Model of Metabolic Evolution: Jasmonate-Responsive Transcription Factors and a Conceptual Model for the Evolution of Metabolic Pathways

**DOI:** 10.3389/fpls.2019.00560

**Published:** 2019-05-14

**Authors:** Tsubasa Shoji

**Affiliations:** Department of Biological Science, Nara Institute of Science and Technology (NAIST), Ikoma, Japan

**Keywords:** alkaloids, *cis*-regulatory element, jasmonates, recruitment model of metabolic evolution, regulon, specialized metabolism, terpenoids, transcription factor

## Abstract

Plants produce a vast array of structurally diverse specialized metabolites with various biological activities, including medicinal alkaloids and terpenoids, from relatively simple precursors through a series of enzymatic steps. Massive metabolic flow through these pathways usually depends on the transcriptional coordination of a large set of metabolic, transport, and regulatory genes known as a regulon. The coexpression of genes involved in certain metabolic pathways in a wide range of developmental and environmental contexts has been investigated through transcriptomic analysis, which has been successfully exploited to mine the genes involved in various metabolic processes. Transcription factors are DNA-binding proteins that recognize relatively short sequences known as *cis*-regulatory elements residing in the promoter regions of target genes. Transcription factors have positive or negative effects on gene transcription mediated by RNA polymerase II. Evolutionarily conserved transcription factors of the APETALA2/ETHYLENE RESPONSE FACTOR (AP2/ERF) and basic helix-loop-helix (bHLH) families have been identified as jasmonate (JA)-responsive transcriptional regulators of unrelated specialized pathways in distinct plant lineages. Here, I review the current knowledge and propose a conceptual model for the evolution of metabolic pathways, termed “recruitment model of metabolic evolution.” According to this model, structural genes are repeatedly recruited into regulons under the control of conserved transcription factors through the generation of cognate *cis*-regulatory elements in the promoters of these genes. This leads to the adjustment of catalytic activities that improve metabolic flow through newly established passages.

## Introduction

Biological processes generally depend on the coordinated expression of multiple genes. Transcription factors play a central role in controlling the RNA polymerase-mediated transcription of downstream genes. These genes form gene networks, or regulons, with transcription factors recognizing specific *cis*-regulatory elements in the promoter regions of these groups of target genes. Complex, often long, metabolic pathways rely on the proper functioning of a large series of metabolic enzymes, membrane transporters, and other proteins. The activity of transcription factors often underlies the coordination of gene expression during various metabolic processes (Patra et al., 2013; Chezem and Clay, 2016; Zhou and Memelink, 2016).

A diverse range of specialized metabolites, such as bioactive alkaloids and terpenoids, are produced and accumulate in various plant species. These metabolites contribute to plant defense and reproduction in a changing environment. Due to their useful attributes, many natural products derived from plants or phytochemicals are utilized as medicines, drugs, dyes, perfumes, or other industrial materials. In contrast to universally present primary metabolites, the occurrence of specialized (or so-called secondary) metabolites is usually restricted to certain taxonomic groups. Metabolite levels are often highly variable, even within a single species or individual plant, reflecting the temporal and spatial dynamics of their production. Genomic and molecular approaches, often involving coexpression network analysis to select candidate genes (Yonekura-Sakakibara and Saito, 2013), have greatly facilitated the identification of structural genes involved in metabolic pathways. By contrast, the regulatory aspects of these genes, such as regulatory mechanisms at the transcriptional and other levels, have remained unexplored, representing a promising area of research in the coming years.

The central roles of MYB and basic helix-loop-helix (bHLH) family transcription factors in regulating the anthocyanin and related flavonoid pathways of many plant species have been a cornerstone example of the persistence of metabolic regulons comprising master transcriptional regulators with their downstream structural genes (Patra et al., 2013; Chezem and Clay, 2016). Well-studied instances include the regulation of the glucosinolate pathway by MYB family factors in *Arabidopsis* (Chezem and Clay, 2016). In notable contrast to the regulators that target a specific metabolic pathway (or set of related pathways), jasmonate (JA)-responsive factors in certain subgroups of the APETALA2/ETHYLENE RESPONSE FACTOR (AP2/ERF) and bHLH families have been found to be master regulators for a diverse range of specialized pathways, mostly for important alkaloids and terpenoids, in distinct plant linages (Zhou and Memelink, 2016).

JAs are phytohormones derived from the octadecanoid pathway that play central roles as signaling molecules during biotic and abiotic stress responses in plants (Goossens et al., 2016a). Many specialized pathways are readily elicited by JA treatment (Goossens et al., 2016a; Zhou and Memelink, 2016). Indeed, many phytochemicals are thought to be involved in plant defense responses against pathogens and herbivores based on the JA-dependent elicitation of their biosynthetic pathways, along with their toxicity to biological agents. The perception of JA signals and the resulting cascades leading to gene regulation primarily occur via proteasome-dependent degradation of JAZ repressor proteins and the subsequent liberation of a few key transcription factors, including bHLH family member MYC2, from JAZ-mediated repression (Thines et al., 2007; Sheard et al., 2010; Zhang et al., 2015; Goossens et al., 2016a). It is important to address how the upstream JA signaling circuit is anchored to downstream defense metabolism. A handful of the JA-responsive transcription factors of AP2/ERF and bHLH families, have been identified as missing links between the highly conserved JA signaling module and more divergent downstream pathways (Zhou and Memelink, 2016).

In this article, I provide an overview of the JA-responsive factors and their target metabolic pathways, which encompass a substantial portion of the specialized pathways for which transcriptional regulators have been defined (Patra et al., 2013; Zhou and Memelink, 2016). The identification of such evolutionarily conserved regulators targeting divergent pathways prompt me to contemplate how these metabolic regulons have been established during the evolution. This evolutionary issue is discussed and a conceptual model is proposed, mainly focusing on the JA-responsive factors and their regulons.

## Clade II, Subgroup IXa ERF Transcription Factors

Transcription factors of the AP2/ERF family are widespread in plants. The GCC-box (5′-AGCCGCC-3′) element is a typical sequence recognized by AP2/ERF transcription factors. The DNA-binding AP2/ERF domain contains a three-stranded β-sheet followed by an α-helix, which form a unique interface required for DNA binding (Allen et al., 1998).

**Figure 1:**
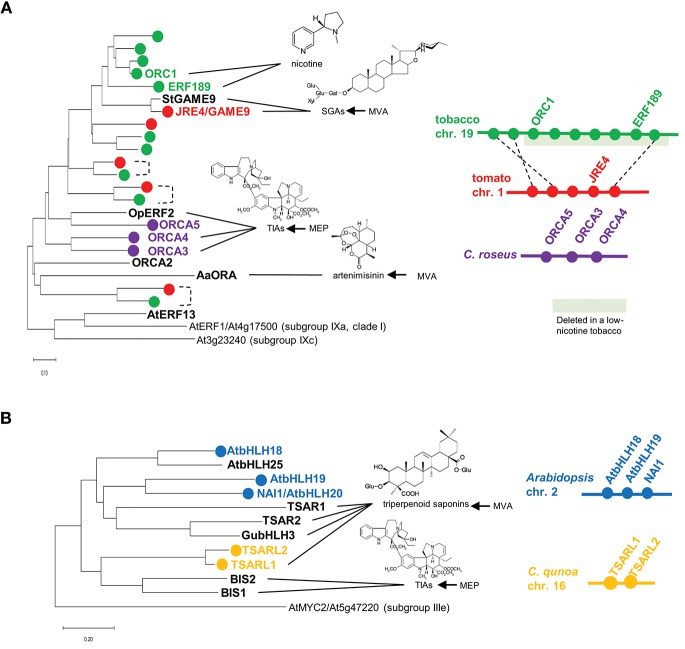
Phylogenetic relationships and gene clustering of JA-responsive ERF **(A)** and bHLH **(B)** transcription factors of clade II, subgroup IXa and IVa, respectively. Full-length amino acid sequences were aligned using ClustalW (Thompson et al., 1994), and the trees were generated with the neighbor-joining algorithm using MEGA-X (Kumar et al., 2018). The *Arabidopsis* genes are included in the trees as an outgroup. The scale bars indicate the number of amino acid substitutions per residue. For isoprenoid-derived metabolites, the contribution of the mevalonate (MVA) or methylerythritol phosphate (MEP) pathway to their production is indicated on the right. Apparent orthologs from Solanaceae plants are connected by dotted lines. Gene clusters are depicted schematically. The region deleted in a low-nicotine tobacco cultivar (Kajikawa et al., 2017) is highlighted in light green.

A group of AP2/ERF family transcription factors, including Octadecanoid-derivative Responsive Catharanthus AP2-domain (ORCA) proteins from *Catharanthus roseus* (Van der Fits and Memelink, 2000; Paul et al., 2017), OpERF2 from *Ophiorrhiza pumila* (Udomsom et al., 2016), ERF189 and ORC1 from tobacco (*Nicotiana tabacum*; Shoji et al., 2010; De Boer et al., 2011), JASMONATE RESPONSIVE ERF4 (JRE4)/GLYCOALKALOID METABOLISM9 (GAME9) from tomato and potato (Cárdenas et al., 2016; Thagun et al., 2016; Nakayasu et al., 2018), and AaORA from *Artemisia annua* (Lu et al., 2013), are classified into clade II of subgroup IXa (Nakano et al., 2006; Shoji et al., 2010, 2013). These transcription factors are involved in regulating JA-mediated defense metabolism in various plants. The JA-responsive *ERF* genes are present in a wide range of eudicots, usually as multicopy genes (Figure 1A). Multiple *ERF* genes are tandemly clustered on chromosomes in some plant genomes (Figure 1A). The phylogenetic relationships of ERFs from different species (Figure 1A) imply that independent generations of these gene clusters in distinct plant families through tandem gene duplication.

### ORCAs in *C. roseus*

Terpenoid indole alkaloids (TIAs) are a large group of specialized products, including the valuable chemotherapy drugs vinblastine and vincristine. A variety of TIAs are derived from the key intermediate strictosidine, which is formed by condensation between tryptamine (a product of the shikimate pathway) and the seco-iridoid compound secologanin. TIA biosynthesis and its regulation have been intensively studied in the medicinally important species *C. roseus* (Apocynaceae) (Zhu et al., 2014). In *C. roseus*, ORCA2 and ORCA3 function as transcriptional regulators that induce the expression of TIA biosynthesis genes, including *strictosidine synthase* and *tryptophan decarboxylase*, encoding key enzymes in this pathway (Menke et al., 1999; Van der Fits and Memelink, 2000; Li et al., 2013). *ORCA3* is physically linked to *ORCA4* and *ORCA5*, forming a gene cluster in the genome (Figure 1A, Paul et al., 2017). ORCA4 shares an overlapping function with ORCA2 and ORCA3, but ORCA4 also targets additional TIA genes (Paul et al., 2017). Unlike *ORCA2* (Li et al., 2013) and *ORCA3* (Van der Fits and Memelink, 2000), the overexpression of *ORCA4* causes a drastic increase in TIA accumulation (Paul et al., 2017). *C. roseus* MYC2 (CrMYC2) directly upregulates the expression of *ORCA3* by recognizing a G-box-like element in its promoter (Zhang et al., 2011), and it also coregulates TIA structural genes with ORCA3 (Paul et al., 2017). In addition to their role in transcriptional regulation, ORCAs and CrMYC2 are activated by phosphorylation by a kinase involved in a JA-activated MAP kinase cascade (Paul et al., 2017).

### OpERF2 in *Ophiorrhiza pumila*

Camptothecin is an antitumor TIA that functions by inhibiting DNA topoisomerase I activity. This clinically important TIA is produced by various angiosperms from taxonomically distant families, including *Ophiorrhiza pumila* (Rubiaceae) (Sirikantaramas et al., 2007). *OpERF2* was originally isolated from *O. pumila* hairy roots. The suppressed expression of this gene resulted in the reduced expression of genes involved in seco-iridoid and upstream methylerythritol phosphate (MEP) pathways, which supply secologanin for downstream camptothecin production, although this did not have a significant impact on TIA accumulation (Udomsom et al., 2016).

### ERF189 and ORC1 in Tobacco

Nicotine is composed with two heterocyclic rings, the ornithine-derived pyrrolidine ring and the nicotinate-derived pyridine ring. In tobacco (*Nicotiana tabacum*, Solanaceae), this toxic alkaloid is produced in roots and primarily accumulates in leaves, functioning as a defense compound against herbivores (Dewey and Xie, 2013). Tobacco *ERF189, ORC1*, and related *ERF* genes are clustered together in the tobacco genome (Figure 1A, Shoji et al., 2010; De Boer et al., 2011; Kajikawa et al., 2017). A cluster of *ERF*s including *ERF189* and *ORC1* were found to be deleted to a large extent in a tobacco cultivar with low nicotine content (Figure 1A, Shoji et al., 2010; Kajikawa et al., 2017). Although not yet proven, ERF189 is considered to be a primary transcriptional regulator of nicotine biosynthesis, given that its expression profiles are similar to those of the downstream biosynthesis genes: strong expression in roots (Kajikawa et al., 2017), no induction in response to NaCl (Shoji and Hashimoto, 2015; Kajikawa et al., 2017), and the suppression of JA-dependent induction by ethylene (Shoji et al., 2000, 2010). A large series of metabolic and transport genes in this pathway are upregulated by ERF189, which recognizes P-box elements, but not the typical GCC-box elements, in their promoters (Shoji et al., 2010, 2013; Shoji and Hashimoto, 2011a). Tobacco MYC2 regulates the expression of *ERF189* and directly activates the transcription of nicotine biosynthesis genes together with ERF189 (Shoji and Hashimoto, 2011b; Zhang et al., 2012).

### JRE4/GAME9 in Tomato and Potato

Steroidal glycoalkaloids (SGAs) are cholesterol-derived, nitrogen-containing metabolites found in the inedible parts of Solanaceae plants such as tomato (*Solanum lycopersicum*) and potato (*S. tuberosum*) (Cárdenas et al., 2015). In the tomato and potato genomes, the *JRE4*/*GAME9* gene is present in a cluster with related *ERF* genes (Cárdenas et al., 2016; Thagun et al., 2016). JRE4/GAME9 regulates nearly an entire series of SGA metabolic steps, including those in the upstream isoprenoid-producing mevalonate (MVA) pathway (Cárdenas et al., 2016; Thagun et al., 2016; Nakayasu et al., 2018). A loss of JRE4/GAME9 function drastically reduced SGA accumulation and resistance to chewing insects in tomato, demonstrating the major role of this transcription factor in defense-related SGA formation (Nakayasu et al., 2018). Tomato MYC2 and JRE4/GAME9 synergistically activated the promoters of SGA genes in tobacco protoplasts (Cárdenas et al., 2016). In agreement with the results of promoter binding studies, cognate *cis*-regulatory elements are significantly enriched in the proximal promoter regions of SGA biosynthesis genes, supporting the direct regulation of these genes by JRE4/GAME9 (Thagun et al., 2016). A comparison of the genomes of ancestral and cultivated species of the *Solanum* genus pointed to the possible selection of certain alleles of *JRE4*/*GAME9* during domestication, which might have contributed to the decrease in antinutritional SGA levels in cultivated *Solanum* species (Hardigan et al., 2017; Zhu et al., 2018).

### AaORA in *Artemisia annua*

Artemisinin, a sesquiterpene lactone produced by the traditional Chinese herb *Artemisia annua* (Asteraceae), has been exploited as an effective anti-malaria agent (Tang et al., 2014). *A. annua* ORA (AaORA) is a transcriptional regulator of artemisinin biosynthesis that upregulates the expression of genes involved in this pathway, including *amorpha-4,11-diene synthase, CYP71AV1*, and *double bond reductase 2* (Lu et al., 2013). *AaORA* is specifically expressed in the trichomes of aerial organs, including artemisinin-producing glandular trichomes (Olofsson et al., 2011; Lu et al., 2013). Since numerous transcription factors from various families (e.g., bHLH, ERF, bZIP, and WRKY), in addition to AaORA, were shown to regulate artemisinin biosynthesis (Tang et al., 2014; Lv et al., 2017), the relative importance of each transcription factor and their functional relationships in this process should be addressed.

### *Arabidopsis thaliana* ERF13

*AtERF13* is the only member of clade II, subgroup IXa in *Arabidopsis thaliana* (Brassicaceae). In contrast to the ERFs mentioned above, AtERF13 was not yet shown to be involved in a specific metabolic pathway. *AtERF13* is induced in response to a range of biotic and abiotic stresses, such as JA, wounding, insect feeding, colonization of beneficial bacteria, high osmolality, and NaCl (Lee et al., 2010; Sogabe et al., 2011; Srivastava et al., 2012; Schweizer et al., 2013). AtERF13 binds to COUPLING ELEMENT1 (CE1), a *cis*-regulatory element required for abscisic acid (ABA)-responsive gene expression, and the overexpression of *AtERF13* confers increased sensitivity to ABA in *Arabidopsis*, suggesting this gene functions in abiotic stress resistance (Lee et al., 2010). AtERF13 is also involved in resistance to insect herbivores, acting downstream of MYC2 (a central player in JA signaling) and mediating the expression of a subset of MYC2-regulated defense genes (Schweizer et al., 2013). AtERF13 is phosphorylated at its tyrosine residues, as revealed by phosphoproteomic analysis, suggesting that its activity is regulated via post-translational modification (Nemoto et al., 2015).

## Subgroup IVa bHLH Transcription Factors

Another group of JA-responsive transcription factors is attracting attention as regulators of metabolic pathways in diverse plants. These transcription factors include bHLH IRIDOID SYNTHESIS1 (BIS1) and BIS2 from *C. roseus* (Van Moerkercke et al., 2015, 2016), TRITERPENE SAPONIN BIOSYNTHESIS ACTIVATING REGULATOR1 (TSAR1) and TSAR2 from *Medicago truncatula* (Mertens et al., 2016a), TSAR-LIKE1 (TSARL1) from *Chenopodium quinoa* (Jarvis et al., 2017), GubHLH3 from *Glycyrrhiza uralensis* (Tamura et al., 2018), and bHLH18, bHLH19, bHLH20/NAI1, and bHLH25 from *Arabidopsis* (Matsushima et al., 2004), which all belong to subgroup IVa of the bHLH family (Figure 1B, Heim et al., 2003; Goossens et al., 2016b).

Unlike AP2/ERF family members, which are specific to plants, the bHLH transcription factor family is widely present in eukaryotic organisms and has expanded, especially in land plants (Feller et al., 2011). The signature bHLH domain is composed of an N-terminal basic region that binds to negatively charged DNA and a helix-loop-helix motif responsible for protein dimerization. bHLH transcription factors form homo- or heterodimers that typically bind to E-box (5′-CANNTG-3′) elements, such as G-box (5′-CACGTG-3′) and N-box (5′-CACGAG-3′) elements, in the promoter regions of their target genes.

### BISs in *C. roseus*

In addition to ORCAs and CrMYC2, BIS1 and BIS2, a pair of homologous JA-responsive bHLH transcription factors, are involved in regulating TIA formation in *C. roseus*. BISs specifically act on a branch of the TIA pathway that supplies the seco-iridoid intermediate, secologanin, for incorporation into TIAs. Overexpression of *BIS1* or *BIS2* results in the upregulation of genes involved in seco-iridoid and upstream MEP pathways, thereby increasing the accumulation of downstream TIAs (Van Moerkercke et al., 2015, 2016). The finding that *BIS2* is induced by *BIS1* or *BIS2* overexpression points to the existence of a positive feedback loop (Van Moerkercke et al., 2016). In contrast to subgroup IIIe MYC2 transcription factors, BISs cannot interact with JAZ proteins and are thus not direct targets of the repressors integrated into the JA signaling module (Van Moerkercke et al., 2016).

### TSARs in *Medicago truncatula*

The model legume plant *Medicago truncatula* (Fabaceae) produces oleanane-type triterpenoid saponins. These amphipathic glycosides, containing triterpenoid aglycones, exhibit a diverse range of biological activities (Osbourn et al., 2011). In *M. truncatula*, TSAR1 and TSAR2, two homologous bHLH transcription factors, are JA-responsive transcriptional regulators of triterpenoid saponin biosynthesis (Mertens et al., 2016a). While the isoprenoid-producing MVA pathway is commonly targeted by both TSARs, TSAR1 and TSAR2 specifically regulate two distinct downstream branches of this pathway, producing nonhemolytic and hemolytic saponins, respectively (Mertens et al., 2016a). TSARs activate the gene encoding 3-Hydroxy-3-Methylglutaryl-CoA Reductase, a rate-limiting enzyme in the MVA pathway, by directly recognizing the N-box element in its promoter (Mertens et al., 2016a).

### TSARL1 in *Chenopodium qunoa*

*Chenopodium quinoa* (Chenopodiaceae), or quinoa, is a staple food crop in Andean countries. Quinoa seeds have high nutritional value, but bitterness of the seeds due to the accumulation of triperpenoid saponins (oleanane-type) is disadvantageous (Kuljanabhagavad et al., 2008). In *C. quinoa, TSARL1* and *TSARL2* are clustered together (Figure 1B) and are expressed in seeds and roots, respectively (Jarvis et al., 2017). In sweet quinoa strains, loss-of-function mutations of *TSARL1*, including one that appears to cause alternative splicing, allowed the down-regulation of genes involved in the production of the antinutritional saponins (Jarvis et al., 2017).

### GubHLH3 in *Glycyrrhiza uralensis*

The medicinal legume *Glycyrrhiza uralensis* (Fabaceae) is rich in oleanane-type triterpenoid saponins, such as glycyrrhizin, which is used as a pharmaceutical compound and sweetener, as well as soyasaponins (Hayashi and Sudo, 2009). *G. uralensis* bHLH3 (GubHLH3), a JA-responsive bHLH transcription factor, upregulates the expression of soyasaponin biosynthesis genes, such as those encoding CYP93E3 and CYP72A566, which are involved in oxidative modifications of the triterpenoid backbone (Tamura et al., 2018). Consistently, the overexpression of *GubHLH3* increased the levels of soyasapogenol B and other intermediates of the soyasaponin pathway in *G. uralensis* hairy roots (Tamura et al., 2018).

### bHLH18, bHLH19, bHLH20/NAI1, and bHLH25 in *Arabidopsis*

In *Arabidopsis*, four genes, *bHLH18, bHLH19, bHLH20/NAI1*, and *bHLH25*, encode subgroup IVa bHLH transcription factors; three of them, except *bHLH25*, form a gene cluster (Figure 1B). *NAI1*, which resides in this three-gene cluster, is indispensable for the formation of the ER body, an ER-derived rod-shape organelle found in plants of the *Brassicales* order (Matsushima et al., 2004). ER bodies are constitutively present in *Arabidopsis* seedlings and roots. By contrast, in rosette leaves, wounding and JA treatment induce the formation of this defense-related organelle, which accumulates large amounts of β-glucosidases, whose activities increase when the compartment is disrupted (Nakano et al., 2014). NAI1 regulates the expression of genes encoding proteins required for ER body formation and activity, including PYK10, a major β-glucosidase in this organelle. PYK10 functions as a myrosinase that hydrolyzes indole glucosinolates, a group of important defense compounds in *Arabidopsis* and related species (Nakano et al., 2017). The phylogenetic co-occurrence of ER bodies and indole glucosinolates and the co-expression of the associated genes also support the functional coordination between this organelle and glucosinolate metabolism (Nakano et al., 2017).

A previous study suggested the involvement of bHLH18, bHLH19, bHLH20/NAI1, and bHLH25 in JA-mediated inhibition of iron uptake in *Arabidopsis* (Cui et al., 2018). JA represses iron uptake by promoting the degradation of FIT/bHLH29, a central transcriptional regulator of iron-uptake genes critical to metal homeostasis (Cui et al., 2018). The four subgroup IVa bHLHs interact with FIT protein and promote its JA-stimulated removal through proteasome-dependent degradation (Cui et al., 2018).

## The Gain of *cis*-Regulatory Elements

The recruitment of metabolic genes into regulons likely requires the gain of transcription factor-binding *cis*-regulatory elements in the appropriate promoter regions. Such a process is fairly likely, considering the relatively frequent, simple generation of short sequence elements in noncoding promoter regions that can have a degree of redundancy and acquire functions through mutational changes, such as point mutations and transpositions (Wray, 2007; Swinnen et al., 2016).

**Figure 2:**
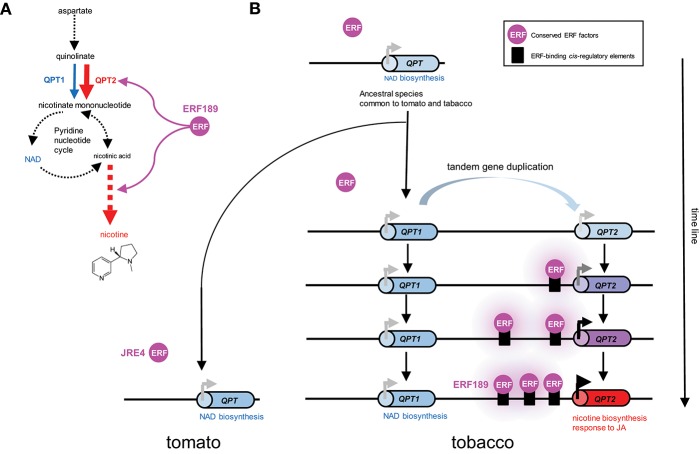
Recruitment of metabolic genes into regulons under the control of conserved transcription factors through the generation of cognate *cis*-regulatory elements in their promoter regions; a case study of the evolution of *QPT2* in the ERF189-controlled nicotine biosynthesis regulon in the tobacco lineage. **(A)** Quinolinate phosphoribosyltransferase (QPT) catalyzes a reaction at the entry point in NAD and nicotine biosynthesis. In tobacco, QPT is encoded by distinct *QPT1* (blue) and *QPT2* (red) genes, which are thought to contribute to NAD and nicotine formation, respectively (Shoji and Hashimoto, 2011a). *QPT2* and downstream steps specific to nicotine formation (red arrows) are regulated by ERF189 in tobacco. Steps including multiple enzymes and undefined reactions are represented by broken arrows. **(B)** Schematic depiction of the evolution of *QPT* genes in the tomato and tobacco lineages. In the tobacco genome, *QPT1* and *QPT2*, which are thought to have arisen through tandem duplication, are located ~75 kb apart on the chromosome. Tomato contains one *QPT* gene copy in a genomic region syntenic to the tobacco cluster (Shoji and Hashimoto, 2019). One of the duplicates, *QPT2*, has become regulated by an evolutionarily conserved ERF transcription factor by gaining *ERF*-binding *cis*-regulatory elements in its promoter. Three functional P-box elements bound by ERF189 are present in the proximal promoter region of *QPT2* in extant tobacco (Shoji and Hashimoto, 2011a).

A case study of a tobacco gene involved in nicotine biosynthesis supports such a scenario. Quinolinate phosphoribosyltransferase (QPT) is a primary metabolic enzyme involved in NAD biosynthesis in all organisms. However, in tobacco, QPT also supplies a significant amount of intermediates required for downstream nicotine biosynthesis (Figure 2A). To satisfy such a metabolic demand, tandem duplication of *QPT* has occurred in the *Nicotiana* lineage, generating a cluster of *QPT1* and *QPT2* genes (Figure 2, Shoji and Hashimoto, 2019). These genes are thought to be involved in NAD and nicotine biosynthesis, respectively, based on their distinct expression patterns (Shoji and Hashimoto, 2011a). *QPT2* harbors multiple ERF189-binding P-box elements in its promoter required for its transcriptional activation by ERF189 (Shoji and Hashimoto, 2011a). The progressive acquisition of these elements after gene duplication has ensured the involvement of *QPT2* in the ERF189-controlled regulon of the nicotine pathway (Figure 2B). The frequent occurrence of JRE4/GAME9 binding elements in the proximal promoter regions of SGA biosynthesis genes in tomato implies that such a notion is also applicable to these genes (Thagun et al., 2016).

## Evolutionary Changes in Transcription Factors

In contrast to the gains (and losses) of *cis*-regulatory elements that strongly contribute to the rewiring of gene regulatory networks, mutational changes in transcription factors, which have profound, pleiotropic effects on numerous downstream genes, are relatively constrained. Nevertheless, there are also examples of the modification of the functionalities and expression patterns of *trans*-acting factors (Maerkl and Quake, 2009).

**Figure 3:**
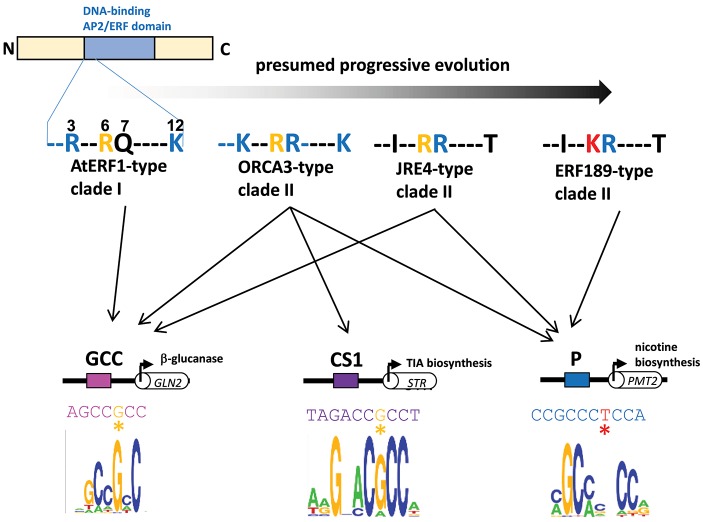
Divergent DNA-binding specificities of subgroup IXa ERF transcription factors. Amino acid residues at positions 3, 6, 7, and 12 in an N-terminal portion of the DNA-binding AP2/ERF domain are shown (counting from the end of the domain). Basic residues R and K at positions 3, 7, and 12 (blue) increase the nonspecific affinity of the proteins for DNA by interacting with negatively charged phosphate backbones (Shoji et al., 2013). The substitution of one residue at position 6 from R (yellow) to K (red) in ERF189-type transcription factors alters the recognition of nucleotide bases (G to T) at a specific position (marked with asterisks) in the *cis*-regulatory elements (Shoji et al., 2013). Perhaps progressive evolution has occurred from ancestral AtERF1-type transcription factors to more specialized ERF189-type transcription factors via functional intermediates with amino acid substitutions at a few positions. The *in vitro* binding specificities of recombinant ORCA3 are represented by sequence logos (Shoji et al., 2013) GLN, β-1,3-glucanase; STR, strictosidine synthase; PMT, putrescine *N*-methyltransferase.

A series of subgroup IXa ERFs have divergent DNA-binding specificities to GCC-box elements and to related but distinct P-box and CS1-box elements. Such distinct binding specificities can be accounted for by a few amino acid differences in a small stretch of the DNA-binding domain (Figure 3, Shoji et al., 2013). It appears that a progressive evolutionary trajectory has led from transcription factors that recognize only a canonical GCC-box to *Nicotiana*-specific ERF189-type transcription factors that bind to P-box but not GCC-box elements via functional intermediates, such as ORCA3-type transcription factors, which bind to multiple elements, including both GCC-box and P-box elements (Figure 3, Shoji et al., 2013). The development of unique combinations of *cis*-elements and *trans*-factors may have been indispensable for avoiding missed connections among unrelated regulatory circuits and, thus, the establishment of lineage-specific specialized pathways. This process appears to have occurred independently of the development of a broad range of ERFs targeting GCC-box elements involved in general defense responses.

Nicotine and SGA biosynthesis pathways in distinct lineages of the same Solanaceae family, which are regulated by orthologous ERFs, share many properties, such as JA-dependent induction and suppression by ethylene (Shoji et al., 2010; Nakayasu et al., 2018). By contrast, the site of their biosynthesis differs between the two lineages: nicotine is synthesized exclusively in tobacco roots, whereas SGAs are produced in nearly all inedible parts of tomato and potato, including leaves and roots. This difference depends on the differential expression patterns of the transcriptional regulators ERF189 and JRE4/GAME9 (Cárdenas et al., 2016; Thagun et al., 2016; Kajikawa et al., 2017). To guarantee the function of each group of metabolites in plant defense, the tissue-specific expression patterns of these regulators may have developed independently after the separation of the two lineages, whereas their responses to JA and other features have been conserved between lineages. These ideas point to the elastic evolution of sets of a particular transcription factor and its downstream metabolic genes as independent units with specialized roles in chemical defense in a lineage-specific manner.

Despite the functional differences noted above, the JA-responsive transcription factors are considered components of conserved regulatory mechanisms present in various species. For instance, in transgenic tomato plants, a promoter reporter of tobacco *QPT2* regulated by ERF189 was expressed in a JA-responsive and cell type-specific manner, as in tobacco, and this expression was mediated by JRE4 (Shoji and Hashimoto, 2019). TSARs and BISs were shown to be functionally exchangeable, as the orthologous bHLH factors regulate each other's target genes in *C. roseus* and *M. truncatula* (Mertens et al., 2016b). Both of these examples clearly point to the interchangeable nature of these factors among species with entirely different pathways (e.g., ornithine-derived nicotine vs. MVA pathway-derived SGAs for ERF189 and JRE4, and MVA pathway-derived saponins vs. MEP pathway-derived TIAs for TSARs and BISs), supporting the functional conservation of these factors.

## Recruitment Model of Metabolic Evolution

**Figure 4:**
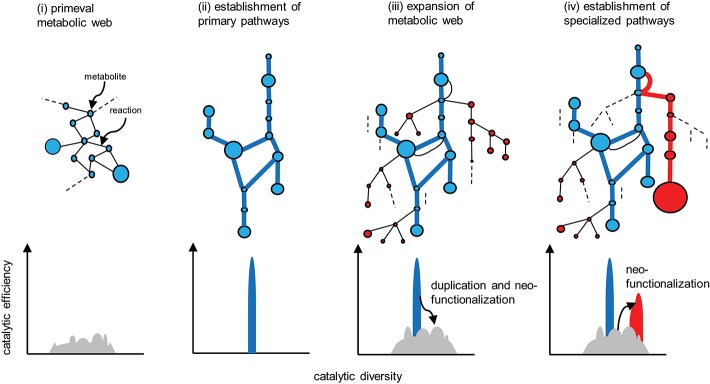
Evolution of metabolic pathways **(upper)** and the associated changes in the catalytic functionalities of the enzymes **(lower)**. Catalytic efficiency (or the activity of a given reaction) and diversity (or the reaction specificity) are represented on the vertical and horizontal axes, respectively. (i) Primeval metabolism depends on low catalytic activities in terms of reaction specificities and efficiencies. (ii) Improved catalytic specificities and efficiencies allow for the emergence of highly efficient primary metabolic pathways (blue lines). (iii) Expansion of the metabolic web occurs via the duplication of metabolic genes, and the subsequent mutational changes in the genes contribute to neofunctionalization of the encoded enzymatic proteins. (iv) A certain series of enzymes are selected and subjected to catalytic improvement (neofunctionalization). Such processes have enabled the emergence of specialized pathways (red lines) that contribute to the substantial accumulation of the metabolites (red circles). Reactions that do not contribute to metabolic flow (and are therefore hidden) are indicated by dotted lines.

Metabolism is a fundamental requirement of all living organisms. Primeval metabolism, or the simple conversion of substances, is thought to rely on a small number of proteinaceous or other catalysts with low reaction specificities and efficiencies (Figure 4i). Metabolic systems have evolved toward increasing order and efficiency (Weng et al., 2012). Contemporary primary metabolism, which was established early and has been maintained, is carried out by robust systems mediated by enzymes with high specificities and efficiencies (Figure 4ii).

Enzymes involved in specialized metabolism are thought to have emerged through duplication, beginning with sophisticated primary enzymes as progenitors, followed by mutational changes in the duplicates and leading to neofunctionalization of the enzymes (Figure 4iii). Relaxed constraints on the specificities and efficiencies of newly generated duplicates allow them to explore a wide range of catalytic possibilities. The promiscuity of multifunctional enzymes, with broader specificities emerging through this process, contributes to the expansion of the metabolic web (Figure 4iii). This web even includes the virtual activities of hidden enzymes (dotted lines in Figure 4) that do not contribute to actual metabolic flow due to limited substrate availability or marginal enzymatic activity; such hidden activities are not readily eliminated by (and are more tolerant to) selection. If these changes are not deleterious or neutral and are thus not eliminated by purifying selection, the metabolic grids continue to build through neutral evolutionary processes such as genetic drift. It seems reasonable that autotrophic plants accumulate low-molecular-weight metabolites derived from photosynthetic assimilates, which usually have antioxidant properties to some extent and are often sequestered in cellular compartments such as vacuoles. These natural products, including those that accumulate in trace amounts, do not necessarily have adaptive significance (Koonnin, 2016).

**Figure 5:**
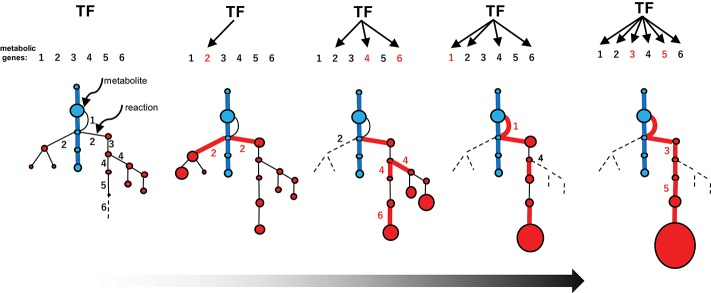
Recruitment model of metabolic evolution. The emergence of an efficient specialized pathway (red lines) from an expanded metabolic web, including the promiscuous steps (part of the web shown in Figure 4iii), is depicted schematically. Metabolic genes and the corresponding steps are numbered (1–6). The recruitment of metabolic genes and enzymes into the regulon under the control of a conserved transcription factor (TF) occurred repeatedly (red numbers). The catalytic specificity of promiscuous enzymes 2 and 4 was optimized (black numbers in the web). The recruitment of genes affecting metabolic flows in the web (which increase the possibility of recruiting other genes into the regulon) and catalytic optimization further enhance favorable flows once established.

The emergence of specialized pathways allowing for the efficient production and accumulation of substantial amounts of metabolites requires the selection of specific flows from the expanded metabolic web, again increasing order and efficiency (Figure 4iv). This process largely relies on positive natural selection rather than neutral evolution, which is dependent on randomness. I propose a conceptual model, recruitment model of metabolic evolution, describing this process. According to this model, structural genes are repeatedly recruited into regulons under the control of evolutionarily conserved transcription factors (which should be activators rather than repressors), such as the JA-responsive ERFs and bHLHs (Figure 5). When a gene in the metabolic web becomes regulated by a transcription factor, obtaining cognate *cis*-regulatory elements, metabolic flows are generated or altered accordingly. Although such events readily occur at high frequency, most of these mutational changes are immediately eliminated and are not maintained in the population. On the other hand, when the newly generated flows result in the accumulation of beneficial products, such as defense compounds, conferring adaptive advantages to the plant, the probability that such changes will be maintained and eventually fixed in the population increases tremendously. Once the beneficial flows occur, the likelihood that mutational events that enhance these flows (such as the transcriptional activation of other metabolic genes and the optimization of catalytic specificities and efficiencies associated with the flows) is expected to rise markedly as well. An initial, mostly accidental, event creating new metabolic flows may trigger cascading mutational changes associated with and improving the flows, eventually leading to the establishment of metabolic regulons and pathways, perhaps within a relatively short evolutionary timescale. The bioinformatic and mathematical bases of the model remain to be explored.

**Figure 6:**
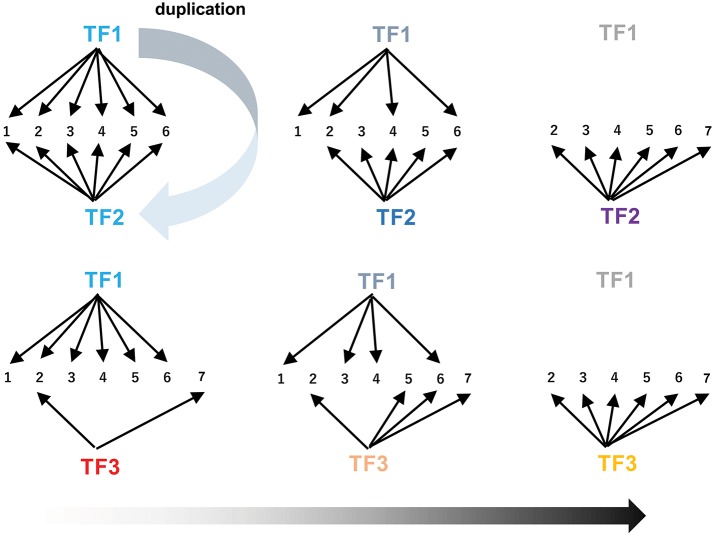
Rewiring of transcriptional circuits. An original regulon (including genes 1 to 6) controlled by TF1 is hijacked by other transcription factors (TF2 and TF3) and eventually modified (removal of 1 and addition of 7). TF2 arose from TF1 through gene duplication. The colors of the TFs reflect functional changes in these proteins during evolution.

The extensive rewiring of transcriptional circuits alters metabolic regulons that were once established under the original transcription factors (Figure 6). During such processes, takeover of the regulons by new transcription factors (including those derived from the original transcription factors by duplication) and the associated changes in the connections in the circuits occur frequently through changes in *cis*-regulatory elements and transcription factors (Figure 6, Johnson, 2017). Contemporary regulatory networks are often complex and include multiple transcription factors, which act as either activators or repressors, and in some cases regulate only specific parts of pathways (e.g., ORCA3 regulates some but not all TIA genes). Extensive rewiring of circuits may contribute to the advent of the complicated regulatory organization found in extant metabolic pathways, which also could account for the fact that few metabolic pathways have simple regulons comprising only a single master regulator and its downstream structural genes.

Analyses of metabolic evolution have emphasized mutational changes to catalytic enzymes (Weng et al., 2012; Moghe and Last, 2015). If metabolic genes are not functionally expressed and no flows are associated with these genes, they are not subjected to positive or purifying selection. The recruitment model, which presumes that transcription factors activate genes prior to changes in catalytic activity, adequately addresses this point as well.

Metabolic evolution is driven by functional changes in catalytic enzymes and changes in the expression patterns of metabolic genes. There appear to be limits to the changes made to the catalytic functionalities of enzymes belonging to a limited number of protein families without hampering the structural and functional stability of protein frames. Therefore, changes in the combinations of metabolic genes with specific temporal and spatial expression patterns might also have significantly contributed to the rise in chemodiversity found in plants. Plants have exploited their limited repertoire of enzymes in a combinatorial manner to produce these diverse compounds.

## Perspectives of “Evo-Meta” Biology

“Nothing in biology makes sense except in the light of evolution” is a famous quotation by Dr. Theodosius Dobzhansky, a Ukrainian-American evolutionary geneticist (Dobzhansky, 1973). The discovery of homeotic genes encoding a group of transcription factors that direct the organization of the body plans of vertebrates and invertebrates has helped elucidate the evolutionarily conserved mechanisms governing development, leading to the rise of Evolutionary Developmental (Evo-Devo) biology. Classic anatomy and embryology, as well as modern developmental biology, share some affinity with evolutionary biology. Paleontology based on fossil evidence is one of the main areas of focus in evolutionary biology.

Chemodiversity of specialized products in plants has been shaped through (and is a product of) evolution. Unfortunately, it is difficult to predict the hues and fragrances of ancient flowers from extinct plants. Nevertheless, the chemodiversity found in extant species and the diverse series of plant genomes, including those yet to be explored, is highly informative (Nakamura et al., 2014). A long period of collection of natural products with divergent chemical structures and biological activities, along with a better understanding of the grouping of biosynthetic pathways and associated enzymes, has led to an awareness of some sort of order behind this chemodiversity. Elucidating the molecular biology behind regulatory factors, such as master transcription factors, that orchestrate these metabolic processes is expected to reveal the universal principles ruling the metabolic processes that produce a diverse range of specialized products. The challenges of “Evo-Meta” (Evolutionary Metabolic) biology aimed at uncovering the origins of this chemodiversity are just beginning.

## Author Contributions

The author confirms being the sole contributor of this work and has approved it for publication.

### Conflict of Interest Statement

The author declares that the research was conducted in the absence of any commercial or financial relationships that could be construed as a potential conflict of interest.
